# A cluster-randomized controlled knowledge translation feasibility study in Alberta community pharmacies using the PARiHS framework: study protocol

**DOI:** 10.1186/2055-5784-1-2

**Published:** 2015-01-12

**Authors:** Meagen M Rosenthal, Ross T Tsuyuki, Sherilyn KD Houle

**Affiliations:** 1 Department of Pharmacy Administration, University of Mississippi, 223 Faser Hall, Post Office Box 1848, Mississippi, MS 38677-1848 USA; 2 University of Alberta, 3rd Floor, Brain and Aging Research Building, Edmonton, Alberta T6G 2M8 Canada; 3 School of Pharmacy, University of Waterloo, 200 University Avenue West, Waterloo, Ontario N2L 3G1 Canada

**Keywords:** Pharmacy, Facilitation, Knowledge translation, Knowledge implementation

## Abstract

**Background:**

Despite evidence of benefit for pharmacist involvement in chronic disease management, the provision of these services in community pharmacy has been suboptimal. The Promoting Action on Research Implementation in Health Services (PARiHS) framework suggests that for knowledge translation to be effective, there must be evidence of benefit, a context conducive to implementation, and facilitation to support uptake. We hypothesize that while the evidence and context components of this framework are satisfied, that uptake into practice has been insufficient because of a lack of facilitation. This protocol describes the rationale and methods of a feasibility study to test a facilitated pharmacy practice intervention based on the PARiHS framework, to assist community pharmacists in increasing the number of formal and documented medication management services completed for patients with diabetes, dyslipidemia, and hypertension.

**Methods:**

A cluster-randomized before-after design will compare ten pharmacies from within a single organization, with the unit of randomization being the pharmacy. Pharmacies will be randomized to facilitated intervention based on the PARiHS framework or usual practice. The Alberta Context Tool will be used to establish the context of practice in each pharmacy. Pharmacies randomized to the intervention will receive task-focused facilitation from an external facilitator, with the goal of developing alternative team processes to allow the greater provision of medication management services for patients with diabetes, hypertension, and dyslipidemia. The primary outcome will be a process evaluation of the needs of community pharmacies to provide more clinical services, the acceptability and uptake of modifications made, and the willingness of pharmacies to participate. Secondary outcomes will include the change in the number of formal and documented medication management services in the aforementioned chronic conditions provided 6 months before, versus after, the intervention between the two groups, and identification of feasible quantitative outcomes for evaluating the effect of the intervention on patient care outcomes.

**Results:**

To date, the study has identified and enrolled the ten pharmacies required and initiated the intervention process.

**Conclusion:**

This study will be the first to examine the role of facilitation in pharmacy practice, with the goal of scalable and sustainable practice change.

**Trial registration:**

Clinicaltrials.gov identifier NCT02191111.

## Background

### The need for pharmacists to play a role in chronic disease management

Patients with diabetes, dyslipidemia, and hypertension are not achieving clinical targets as outlined in clinical practice guidelines [1–6]. It is well established that suboptimal control of these conditions increases patients’ risk of major complications such as myocardial infarction, heart failure, stroke, and kidney disease. As these conditions are primarily controlled using medications, adherence to prescribed therapies and appropriate dosing of these therapies become integral in helping to ensure patients achieve optimal outcomes.

Recent evidence from a number of systematic reviews [7–13] suggests that pharmacists’ management of patients’ medications yields improved use of, and adherence to, therapies. Additionally, the recent regulation of pharmacy technicians in Canada allows them to perform the final check on dispensed prescriptions [14]. This has created an opportunity for the greater delegation of this task, thereby freeing pharmacists to focus more specifically on patient care.

### What is holding pharmacists back?

Despite evidence of benefit and greater task flexibility, pharmacists are not providing medication management to all eligible patients with chronic conditions. Research into the reasons for this gap in the treatment of patients points to a number of barriers including a perceived lack of knowledge [15], confidence [16], time, and privacy [16, 17]. Some work also suggests that pharmacists are tied to traditional dispensing models of practice [15, 18, 19], and that the physical environment and workflow of the pharmacy are mainly dispensing-oriented [20].

Previous research into pharmacy practice change has focused on encouraging individual practitioners to drive the change [21], while failing to account for the influence of organizationally erected barriers within community pharmacy. A further assumption of this previous research has been that pharmacists possess the skills needed to identify and address these barriers, revealing what Rogers has called an individual-blame bias [22]. Pharmacists across Canada are interested in providing clinical services to their patients [18], but previous work in this area has neglected to provide instruction for how pharmacists can implement these changes.

### The Promoting Action on Research Implementation in Health Services framework

The Promoting Action on Research Implementation in Health Services (PARiHS) framework proposes three interrelated components as part of knowledge translation strategies: evidence, context, and facilitation [23, 24]. Each of these components must be optimized to ensure the successful implementation of evidence into practice and share a dynamic and simultaneous relationship with each other [25]. According to Kitson and colleagues [23], the most successful implementation occurs when the following criteria are met:

-  *Evidence* is scientifically robust and matches professional consensus and patient preferences.
-  The *context* is receptive to change, including sympathetic workplace culture, strong leadership, and appropriate monitoring and feedback mechanisms.
-  *Facilitation* is provided to make the change process easier for group members, with input from skilled external and internal facilitators.

### Study objective/hypothesis

This paper outlines the protocol for a feasibility study of a facilitated pharmacy practice intervention based on the PARiHS framework. This intervention is intended to assist community pharmacists to increase the number of formal and documented medication management services completed for patients with diabetes, dyslipidemia, and hypertension, within a medium-to-large retail pharmacy chain organization. A cluster-randomized design will be utilized, as the knowledge translation intervention proposed may result in systemic changes in how pharmacies operate, thereby increasing the risk of contamination of the control group if a patient-level randomization were applied.

The objective of the study is to identify the needs of community pharmacies in implementing clinical services into practice, explore the acceptability and utility of facilitation in community pharmacy practice, and conduct a preliminary analysis on the outcomes of the intervention related to the number of services provided and patient care outcomes achieved. Should this feasibility study identify benefit, future work will involve the scale-up of the intervention across a greater number of pharmacies, with the ultimate goal of improving clinical care services provided to patients of community pharmacies.

## Methods/design

### Design

This will be a feasibility study with a cluster-randomized before-after design comparing ten pharmacies from within a single organization, with the unit of randomization as the pharmacy. Pharmacies will be randomized to facilitated intervention, based on the PARiHS framework, or usual practice in a 1:1 ratio. Ethics approval for the project has been obtained from the Health Research Ethics Board at the University of Alberta. Due to the nature of the intervention, blinding is not possible.

### Setting

The study will be conducted in community pharmacies (belonging to the same chain) in the province of Alberta, Canada. Our team has decided to work with a single organization to further ensure baseline consistencies among pharmacies, particularly relating to organizational structure, policies, and job descriptions.

### Participants

#### Pharmacists

Ten pharmacies belonging to the same pharmacy chain within the province of Alberta, Canada, will be identified for participation. Sites must have an interest in, and the ability to provide, medication management services but have not fully integrated these activities. No limitations on prescription count, location, or staffing level were applied. Rather, pharmacies were contacted and invited to participate until consent was received from the staff of ten pharmacies.

#### Patients

All Alberta residents eligible for the medication management services defined below can receive the services and will be counted towards the study outcomes. Intervention pharmacies will be encouraged to employ case finding strategies [26] to identify possibly eligible patients with diabetes, hypertension, and/or dyslipidemia. Case finding strategies use demographic information, as well as knowledge of condition-specific risk factors and symptoms to determine whether or not an individual would benefit from further testing or intervention [26]. By taking this approach to the identification of patient participants, scarce health resources will be saved as wholesale screening of the entire population of patients attending a particular pharmacy will be avoided.

### Randomization

The ten participating sites will be matched by an investigator not involved in enrollment or application of the intervention, into five most similar pairs based on geographic location, prescription volume, staffing level, and the existence of any specialty-certified pharmacists (e.g., Certified Diabetes Educators). However, prescription volume will form the primary matching variable, as time to conduct patient care services among other pharmacy activities has been identified as a key barrier preventing greater performance of these activities in North American pharmacy practice [27–29]. Using a random-number generator, one pharmacy from each pair will then be randomly allocated to either the intervention or control group. All regular staff of each pharmacy constitutes the members of that clustered site. Each regular staff member provided informed consent to participate prior to randomization.

### Intervention

For the purposes of this project, it will be assumed that the evidence component of the PARiHS framework for the value of pharmacists’ intervention in the care of patients with diabetes, dyslipidemia, or hypertension is sufficiently optimized [7–13] and that pharmacists are aware of, and support, this evidence [18, 30].

External, task-focused facilitation [31], informed by an evaluation of contextual factors using the Alberta Context Tool (ACT), on-site observations, and participant interviews, will be applied to train community pharmacies to develop alternative team processes that enable a greater number of medication management services to be provided to patients with pre-specified chronic conditions [32]. Permissions to utilize the ACT have been received. This facilitation process will assist pharmacy team members in thinking through the change process and provide them with a set of skills to develop change strategies in the future. Facilitators will also serve as a liaison between management and front-line staff to implement potential organizational change processes to ensure alignment between expected service provision and the operational reality of pharmacy practice.

The facilitation process will begin with an evaluation of the contexts of all of the pharmacies using the ACT. Then, baseline rates of the completion of medication management services will be measured using pharmacy software. Facilitated discussions (i.e., participant interviews) will follow with pharmacy team members (managers, pharmacists, and technicians) to explore the scores of each of the dimensions on the ACT, and to develop strategies to address any barriers identified. All intervention pharmacy team members will also be asked to complete a facilitation evaluation survey.

From this, an alternative team process will be developed to provide pharmacy teams with guidelines for how more medication management services could be completed. These guidelines may include physical layout considerations, workflow improvements, patient identification strategies including case finding [26], task delegation from pharmacists to pharmacy technicians and assistants [33], and alignment of job description expectations between front-line staff and management.

### Control

Control pharmacies will also complete the ACT, and baseline rates of the completion of medication management services will be collected. However, investigators will not meet with staff from these locations to discuss the results of the ACT or provide any assistance with implementing medication management services. A number of recent changes affecting the practice of pharmacy in Alberta, including simplification of the application process for prescribing authorization [34] and the launch of programs by advocacy organizations to support applying pharmacists [35], the regulation of pharmacy technicians [14], and changes to generic drug pricing and allowable professional fees [36], have occurred. Therefore, the use of a concurrent control group aims to isolate the effect of the intervention on the outcomes of interest from these compounding factors.

### Outcomes

All outcomes measured will be analyzed at the cluster level.

#### Primary outcome

As a feasibility study, process evaluation will form the primary outcome [37]. Process evaluation will be evaluated using the comparative baseline results of the ACT [38–40] for both groups, transcriptions of interviews, field notes, and the facilitation evaluation surveys [41]. These surveys will be undertaken pre- and post-intervention and are designed to capture stakeholders’ expectations, and final thoughts, about the process. Field notes will be kept during site visits and all subsequent interactions with the intervention sites, with the intention of tracking the identified needs, and implementation of the facilitated intervention. Overall, these data are intended to detail the facilitated intervention development process to gain insight into the needs of community pharmacies, the acceptability and uptake of modifications made, and the willingness of pharmacies to participate.

#### Secondary outcomes

**Service provision** To gauge the effectiveness of the intervention in improving medication management service provision rates, secondary outcomes will include descriptive analysis of the change in the number of formal and documented services in the aforementioned chronic conditions provided 6 months before versus after the intervention between the two groups. While the definition of medication management services varies in the literature, for the purposes of this study, they are defined as those services currently eligible for remuneration under the Alberta Pharmacy Services Framework [42] and include the following:

-  Comprehensive Annual Care Plan (CACP)
-  Standard Medication Management Assessment (SMMA)
-  Follow-ups to CACP/SMMA
-  Assessment and adaptation of a prescription
-  Patient assessment for prescription renewal
-  Patient assessment in a medication-related emergency (emergency prescribing)
-  Optional services (those that can only be provided by pharmacists with additional certifications):

○ Assessment and administration of medications by injection

○ Patient assessment for initiating medication therapy

For the purpose of this study, service counts will be limited to the provision of a CACP, SMMA, or follow-ups to CACP or SMMA.

A CACP is designed to meet the unique needs of patients with complex health issues [42]. According to the Alberta Health Pharmacy Services Framework, ‘complex needs’ are defined as patients having at least two of the pre-identified chronic conditions or patients with at least one pre-identified chronic condition and one pre-identified risk factor (see Table 1) [42]. A CACP includes the completion of a patient assessment and a best possible medication history. This information is then used to develop a specific care plan to identify and resolve existing or potential drug-related problems, mutually develop therapy goals, and to follow-up and monitor the progress on identified goals. All information contained within the CACP is formally documented and provided to the patient and any other health professionals involved in the patient’s care, as needed. Prior to conducting a CACP, informed consent from the patient must be obtained.

**Table 1:** **Qualifying conditions and risk factors**

Conditions	Risk factors
Hypertension	Obesity
Diabetes mellitus	Addictions
Chronic obstructive pulmonary disease	Tobacco
Asthma	
Heart failure	
Ischaemic heart disease	

An SMMA is designed to meet the needs of patients who do not qualify for a CACP [42]. In particular, patients must have at least one of the pre-specified chronic conditions and be taking at least three different Schedule 1 (prescription) medications or insulin, have diabetes and be taking at least one Schedule 1 drug or insulin, or use a tobacco product daily and be willing to receive tobacco cessation services. Steps for completing and documenting a SMMA are the same as those for a CACP. Patients having either a CACP or SMMA completed are eligible for follow-up interactions as required and outlined in the care plan developed with the pharmacist [42].

### Identification of feasible patient care outcomes

With the ultimate goal of improving patient care, this study also aims to identify feasible clinical outcomes for collection and analysis, including changes in clinical measures specific to diabetes, hypertension, and dyslipidemia management as recorded on applicable patients’ follow-up documentation. Potential outcome measures may include, but are not limited to the following:

-  Glycosylated hemoglobin (HbA1c)
-  Fasting plasma glucose
-  Systolic and diastolic blood pressure
-  Low-density lipoprotein cholesterol (LDL-C)
-  High-density lipoprotein cholesterol (HDL-C)
-  Triglycerides
-  Total cholesterol to HDL-C ratio

Pharmacy staff and management will be consulted to determine if additional outcomes can be reasonably collected and are relevant for clinical and/or administrative purposes.

### Description of participating pharmacies

To determine the generalizability of this study’s findings to other pharmacies within the chain and externally, descriptive data on the pharmacies will be identified and summarized, including location, prescription count, and staffing level. This data, along with service provision counts and clinical outcomes, will be utilized in the estimation of the intracluster correlation coefficient.

### Data collection

Process evaluation data will be collected over the course of the study. Results from the ACT will be collected at baseline and 6 months, as well as results from the facilitation evaluation surveys for intervention pharmacies. Interview data will be collected as they are conducted through the start-up phase of the intervention, and field notes will be collected continually as data collection for the primary outcome begins. All data will be stored securely at the University of Alberta Epidemiology Coordinating and Research (EPICORE) Centre.

Data on the change in the number of medication management services provided will be collected from pharmacy software programs located within the respective stores participating. Baseline numbers of medication management services provided will be counted back 6 months from the time of data collection (for example, if data collection begins May 1, 2014, pre-intervention data will be collected starting from November 1, 2013). Final data regarding the number of medication management services provided following the intervention will be collected for both intervention and control pharmacies for 6 months starting after the first set of in-person visits with the intervention pharmacies.

Data for the secondary clinical outcomes at baseline and 6 months will be collected at the same time as follow-up data for the primary outcome, for all patients receiving CACP or SMMA services. It is of key importance to note that pharmacist participants will not be asked to complete any data collection for the sole purposes of completion of the study. As the intention of this project is to improve the sustainability of clinical intervention provision by pharmacists, it is paramount that their actions herein are as close as possible to what they would be performing after the conclusion of the study. All clinical parameters will be reported by the pharmacies to the study investigators in aggregate form, without any patient identifying information. These values, and the frequency of their collection, will be analyzed descriptively to gauge whether potentially clinically meaningful changes can be reasonably assessed by this method.

### Statistical analysis

All analyses for the primary and secondary outcomes will be based on the intention to treat principle. Pharmacy information, such as average weekly prescription count or pharmacists with additional certifications, will be presented descriptively for the purpose of determining the generalizability of the findings across all pharmacies in Alberta.

Results from the interviews, field notes, and the facilitation evaluation surveys will be analyzed for the purposes of a formative evaluation of the process of implementation of the facilitated intervention using a grounded theory approach [43]. In the case of this project, grounded theory is intended to develop a theory for the process of implementation of the facilitated intervention within the pilot group of pharmacies. Data analysis will be an ongoing process, as the constant comparative analytic technique will be employed to generate theory and adapt to the needs of the pharmacies, as the intervention is implemented [43]. The process evaluation themes identified by Grant et al*.*[44] for cluster-randomized trials of complex interventions will form the basis of this analysis. Facilitators will also track their time spent interacting with sites and implementing agreed-upon changes, for the purpose of determining the feasibility of providing the service to a larger number of pharmacies for future implementation projects.

We will also include, as part of the feasibility analysis, estimation of the effect size for the difference in change in the number of clinical services provided before and after the intervention between intervention and control groups, along with confidence interval estimates. This information, along with the acceptability of the intervention by pharmacies and members of the pharmacy chain management team and mean amount of facilitator time required per intervention site, will enable us to determine whether scale-up of the intervention across the entire chain is feasible, and if the potential costs to employ a facilitator for this service is likely to be sufficiently offset by increased billing for clinical services from the organization’s perspective.

Where patients received initial and/or follow-up services over the course of the intervention period, counts of the number, and type of clinical outcomes documented will be presented, along with mean baseline and follow-up values for these parameters. Due to the low sample size of this study and the primary focus on process outcomes, hypothesis testing on these values will not be performed.

### Sample size

For this feasibility study, a pre-determined sample size of ten pharmacies (five intervention and five control) was decided upon as this was felt to be manageable by a single volunteer facilitator, and within the budgetary restrictions of our funding. The enrollment of five intervention sites is believed to be sufficient to initially detect common systemic organizational changes that may be of benefit to the pharmacy chain as a whole, while also allowing for refinement of the facilitation process for subsequent scale-up efforts. As the first study of this type in community pharmacies, the intracluster correlation coefficient (ICC) is unknown. A cluster-randomized trial of community pharmacies in Saskatchewan, Canada, estimated an ICC of 0.0143 [45]; however, it must be noted that data utilized to determine this value originated from various types of pharmacies (i.e., department mass-merchandise, chain-franchise, independent) [46]. One would expect our study to identify a higher ICC than reported in previous work due to our utilization of a single pharmacy chain for this study.

## Discussion

The current pharmacy services funding model [42], and recent budget cuts [47], mean that it is increasingly important for pharmacists to demonstrate their value in improving the care of Albertans. It is well established that pharmacist-provided care is clinically effective in improving chronic disease control, but greater provision of these services in community pharmacies is required. The intervention outlined in this pilot study offers an approach to achieving this objective by linking existing evidence to a scalable and sustained change that will then be expanded to other pharmacies.

## Trial status

The study has recruited and enrolled all ten pharmacies and is currently applying the intervention to the pharmacies randomized to facilitation. It is anticipated that the intervention and data collection phases will be completed in 2015.

## Authors’ information

MR is a sociologist and an assistant professor at the Department of Pharmacy Administration at the University of Mississippi. SH is a pharmacist and an assistant professor at the School of Pharmacy at the University of Waterloo. RT is a pharmacist and professor at the Department of Medicine at the University of Alberta.

## Abbreviations

ACT: Alberta Context Tool
CACP: Comprehensive Annual Care Plan
HDL-c: high-density lipoprotein cholesterol
HgbA1c: hemoglobin A1c (glycosylated hemoglobin)
LDL-c: low-density lipoprotein cholesterol
PARiHS: Promoting Action on Research implementation in Health Services
SMMA: Standard Medication Management Assessment.
